# Dataset of histone H3K4me2 modified genes in the liver of female Sprague-Dawley rats with chronic antipsychotic drugs of olanzapine or clozapine

**DOI:** 10.1016/j.dib.2025.111425

**Published:** 2025-02-22

**Authors:** Yueqing Su, Jiamei Lian, Chao Deng

**Affiliations:** a Fujian Maternity and Child Health Hospital, Affiliated Hospital of Fujian Medical University, Fuzhou, China; b College of Clinical Medicine for Obstetrics & Gynaecology and Paediatrics, Fujian Medical University Fuzhou, China; c School of Medical, Indigenous and Health Sciences, and Molecular Horizons, University of Wollongong, Wollongong, NSW 2522, Australia

**Keywords:** Olanzapine, Clozapine, Second generation antipsychotic drugs, ChIP-Sequencing, H3K4me, Liver

## Abstract

Epigenetic histone modifications have been found to be associated with the development of metabolic disorders. However, the potential contribution of these modifications to metabolic disturbances induced by chronic treatment with second-generation antipsychotic drugs (SGAs) remains unclear. This dataset presents chromatin immunoprecipitation (ChIP) data on H3K4me methylation in hepatic tissue of rats treated with olanzapine or clozapine for 9 weeks for a comprehensive view of the effects of SGAs on H3K4me methylation in an animal model. It offers new insights into the epigenetic mechanisms underlying SGAs-induced metabolic side effects.

Specifications Table

**Table:** 

Subject	Epigenetics
Specific subject area	Histone methylation and antipsychotic drugs
Type of data	Tables, Fig.s
Data collection	Female Sprague-Dawley rats were treated for 9 weeks with olanzapine (3 mg/kg, b.i.d, orally, Zyprexa, Eli Lilly, Indianapolis, USA), clozapine (20mg/kg, b.i.d, orally, Clozaril, Novartis, Turkey), or a vehicle (equivalent sweet cookie dough pellet without drugs). Two hours after the final drug treatment, all animals were sacrificed, and their liver were dissected. After shearing to an average fragment length of 200–1,000 bp, the hepatic cross-linking DNA was incubated with chip-grade anti-dimethyl-H3-K4 antibody. The chromatin immunoprecipitation (ChIP) DNA was then used to construct a DNA paired-end library at Novogene Co. (Hong Kong) and performed high throughput sequencing using an Illumina Hiseq2500 50SE sequencer. Quality control (QC) was performed at each step, including sample testing, library preparation, and sequencing, to ensure the accuracy and reliability of the data. The raw reads from the Illumina HiSeqTM X TEN underwent QC to determine their suitability for subsequent analysis and were filtered to obtain clean reads. The clean reads were compared to the Rattus norvegicus reference genome Rnor_6.0 using BWA comparison software. Unique positions on the alignment were extracted for read analysis and peak scanning.
Data source location	Institution: School of Medical, Indigenous and Health Sciences, University of Wollongong City/Town/Region: Wollongong Country: Australia
Data accessibility	Repository name: National Center for Biotechnology Information (NCBI) Data identification number: GSE288060 Direct URL to data:https://www.ncbi.nlm.nih.gov/geo/query/acc.cgi?acc=GSE288060
Related research article	1. Yueqing Su, Xuemei Liu, Jiamei Lian, Chao Deng. Epigenetic histone modulations of PPARγ and related pathways contribute to olanzapine-induced metabolic disorders. Pharmacological Research 2020 May: 155:104703

## 1 Value of the Data

• This data will provide a comprehensive genome-wide view of the effects of second generation antipsychotics on H3K4me methylation.
• This dataset will offer new insights into the epigenetic mechanisms underlying antipsychotic-induced metabolic side effects.
• This unique profile of H3K4me methylation in olanzapine or clozapine treated animals will help explain the differences in their observed metabolic characteristics.

## 2 Background

Both animal and human studies suggested that antipsychotic drugs could alter epigenetic homeostasis and induce pharmacoepigenomic effects, indicating a promising role for these drugs in modulating the host epigenome [[1], [2], [3]]. Histone H3 lysine 4 (H3K4me3) is associated with transcriptional start sites and is proposed to regulate transcription initiation [4]. It may also serve as a metabolic sensor [5]. However, the epigenetic mechanisms involving H3K4me methylation that underlie metabolic disorders induced by antipsychotic treatment remain poorly understood.

Chromatin immunoprecipitation followed by next-generation sequencing (NGS) (ChIP-seq) is the most widely used method for analysing genome-wide DNA-protein interactions, which explains the genomic distribution of chromatin-associated proteins, histone posttranslational modifications, and histone variants. This enables systematic analysis of how the epigenomic landscape contributes to disease [6,7].

Thus, to investigate whether chronic treatment with olanzapine and clozapine has differential effects on histone H3K4me modification, ChIP-seqanalysis was performed on hepatic tissue dissected from rats after 9 weeks’ drug administration.

## 3 Data Description

### 3.1 Data quality control

#### 3.1.1 Distribution of sequencing quality

**Fig. 1:**
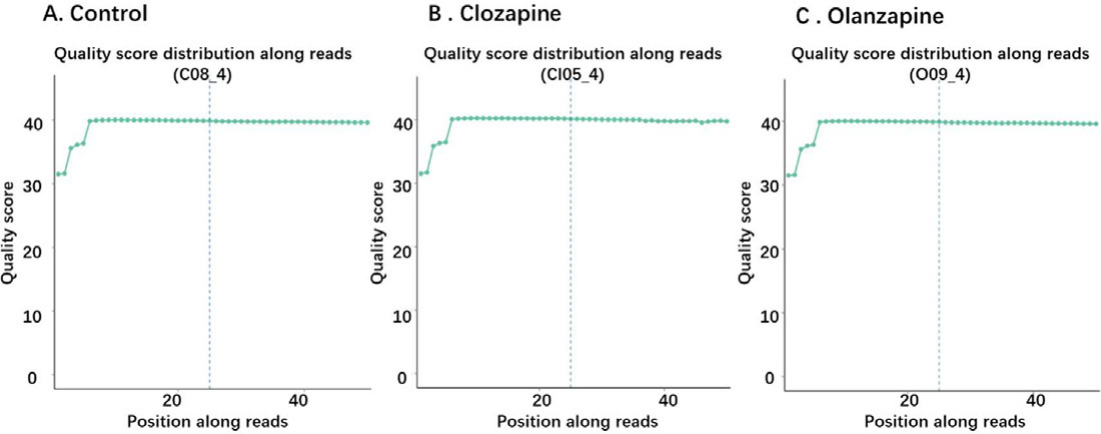
Distribution of Sequencing Quality. **A**. Control, **B**. Clozapine, **C**. Olanzapine. The base position is on the horizontal axis, and the sequencing quality is on the vertical axis.

The “e” represents the sequence error rate, and Qphred represents the base quality value, calculated as Qphred=-10log10(e). The relationship between sequencing error rate (e) and sequencing base quality value (Qphred) is presented in Table 1. The distribution of quality score is shown in Fig. 1.

**Table 1:** The relationship between sequencing error rate (e) and sequencing base quality value.

Phred score	error base	right base	Q-score
10	1/10	90%	Q10
20	1/100	99%	Q20
30	1/1000	99.9%	Q30
40	1/10000	99.99%	Q40

#### 3.1.2 Distribution of sequencing error rate

**Fig. 2:**
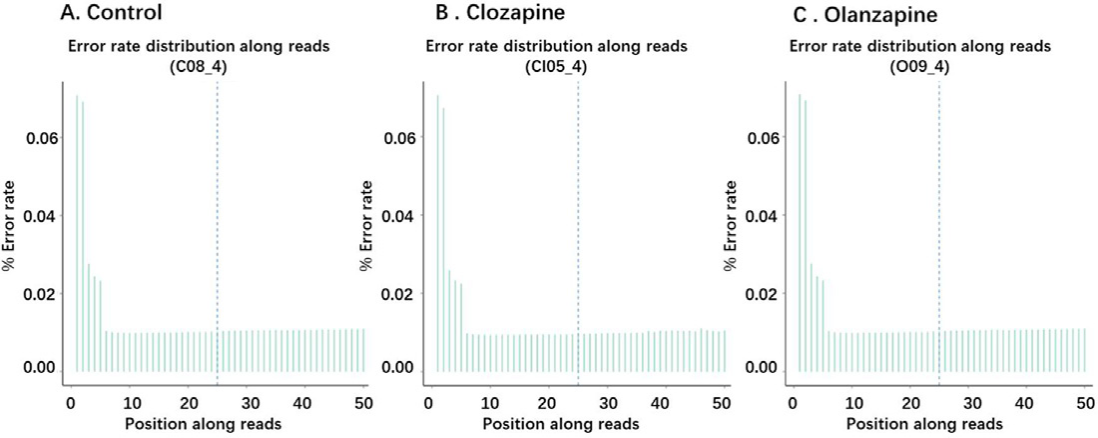
Error Rate Distribution. **A**. Control, **B**. Clozapine, **C**. Olanzapine. The base position is on the horizontal axis, and the single base error rate is on the vertical axis.

The error rate of this project is shown in Fig. 2.

#### 3.1.3 Distribution of A/T/G/C base

According to the principle of complementary bases, the content of AT and GC should be equal at each sequencing cycle and remain constant and stable throughout the sequencing process. However, in practical measurements, due to the primer amplification bias and other factors, fluctuations in the first 6 to 7 nucleotides are normal and reasonable.

**Fig. 3:**
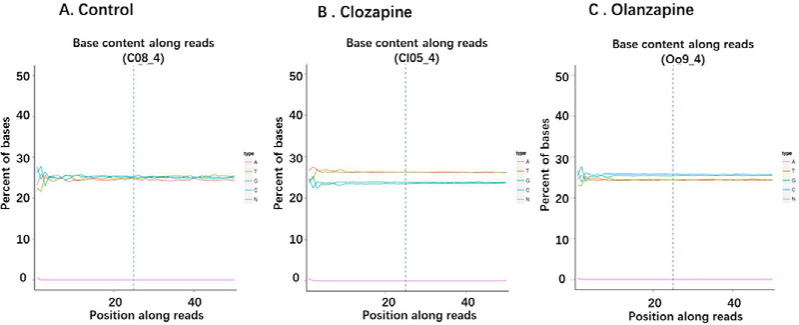
A/T/G/C Distribution. **A**. Control, **B**. Clozapine, **C**. Olanzapine. The base position is on the horizontal axis, and the single base percentage is on the vertical axis.

The distribution of GC content is shown in Fig. 3.

#### 3.1.4 Results of raw data filtering

**Fig. 4:**
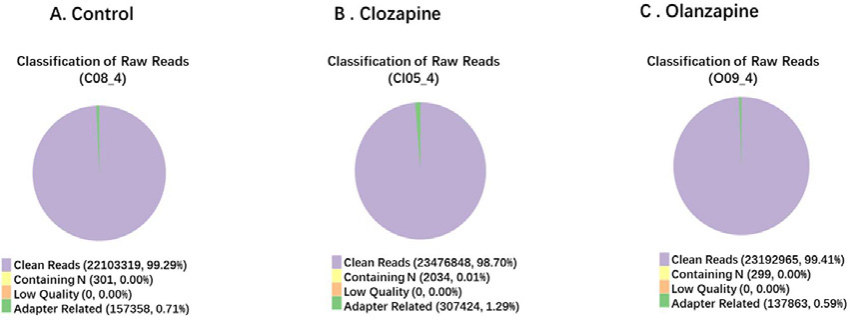
Composition of Raw Data. **A.** Control, **B.** Clozapine, **C.** Olanzapine. Different color for different components: (1) Purple: Adapter related: (reads containing adapter) / (total raw reads); (2) Yellow: Containing N: (reads with more than 10% N) / (total raw reads); (3) Orange: Low quality: (reads of low quality) / (total raw reads); (4) Green: Clean reads: (clean reads) / (total raw reads).

The Sequencing data filtration of this project can be seen in Fig. 4.

### 3.2 Summary of sequencing data information

#### 3.2.1 The data filtered from raw data: clean data 10.4 G

**Fig. 5:**
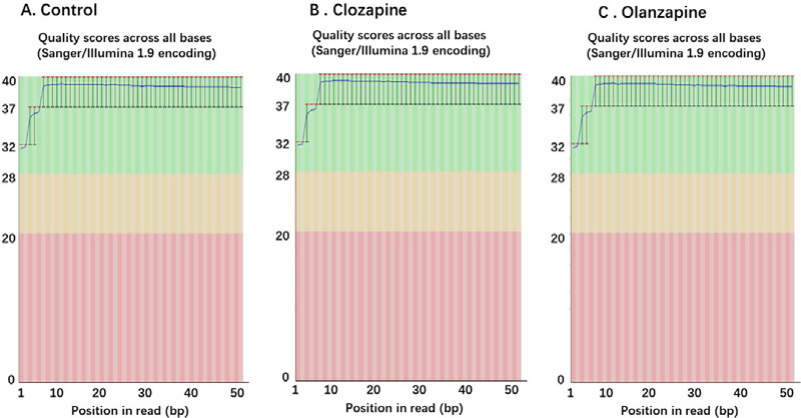
Bases distribution of filtered reads in each sample. **A**. Control, **B**. Clozapine, **C**. Olanzapine. The horizontal coordinate is the position of the base of read, and the vertical coordinate represents the mass value of the base at the corresponding site of reads.

The detail statistics for the quality of sequencing data are shown in Table 2. Bases distribution of filtered reads in each sample is presented in Fig. 5.

**Table 2:** Data quality summary.

Sample	Raw Reads	Clean Reads	Raw Base(G)	Clean Base(G)	Effective Rate (%)	Error Rate (%)	Q20(%)	Q30(%)	GC Content (%)
clo5_4	23786306	23476848	1.2	1.2	98.7	0.01	99.17	97.38	50.45
co8_4	22260978	22103319	1.1	1.1	99.29	0.01	99.01	96.74	50.49
o09_4	23331127	23192965	1.2	1.2	99.41	0.01	99	96.72	51.17

Note: Raw reads: total amount of reads of raw data, each four lines taken as one unit. For paired-end sequencing, it equals the amount of read1 and read2; otherwise, it equals the amount of read1 for single-end sequencing. Clean reads: total amount of reads of clean data, each four lines taken as one unit. For paired-end sequencing, it means the amount of read1 and read2; otherwise, it equals the amount of read1 for single-end sequencing. Raw bases: (Raw reads) * (sequence length), calculating in G. For paired-end sequencing like PE150, sequencing length equals 150; otherwise, it equals 50 for sequencing like SE50. Clean bases: (Clean reads) * (sequence length), calculating in G. For paired-end sequencing like PE150, sequencing length equals 150; otherwise, it equals 50 for sequencing like SE50. Effective Rate (%): (Clean reads/Raw reads) *100%. Error rate: base error rate. Q20, Q30: (Base count of Phred value > 20 or 30) / (Total base count). GC content: (G & C base count) / (Total base count).

#### 3.2.2 Genome comparison analysis

The outcomes of genome comparison analysis are presented in Table 3.

**Table 3:** Results of genome comparison analysis (rattus_norvegicus reference genome Rnor_6.0 (http://asia.ensembl.org/Rattus_norvegicus/Info/Index).

Sample	genome	upstream 5kb	UTR5	Exons	Introns	UTR3	downstream 5kb
	reads (%)	reads (%)	reads (%)	reads (%)	reads (%)	reads (%)	reads (%)
c08_4	8587304(100)	332773(3.88)	122228(1.42)	191338(2.23)	2307882(26.88)	37262(0.43)	169194(1.97)
cl05_4	2076477(100)	58798(2.83)	41930(2.02)	99700(4.80)	489441(23.57)	21600(1.04)	47988(2.31)
O09_4	10203016(100)	695375(6.62)	263804(2.59)	413489(4.05)	3307512(32.42)	54874(0.54)	197752(1.94)

#### 3.3.3 Identification of binding loci

**Fig. 6:**
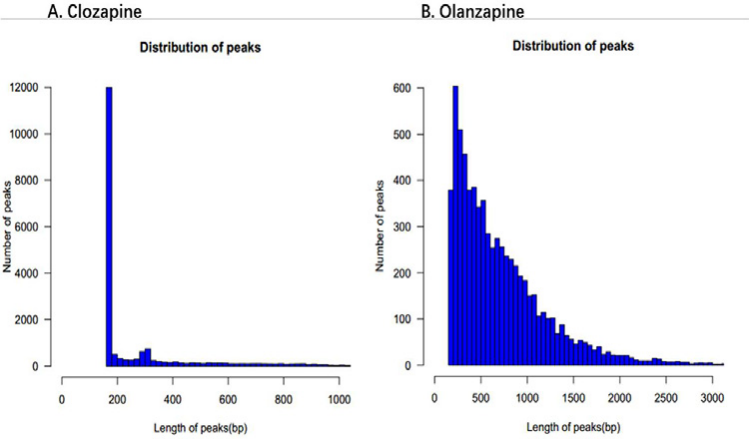
Distribution of peak. **A.** Clozapine, **B.** Olanzapine.

**Fig. 7:**
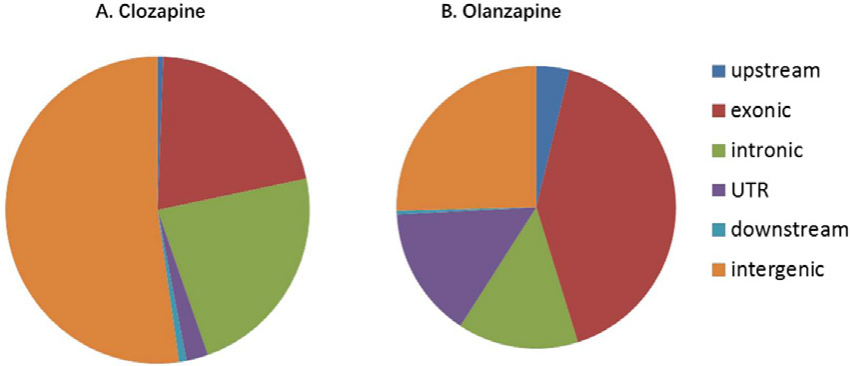
Distribution of binding gene functional elements. **A.** Clozapine, **B.** Olanzapine.

The distribution of peaks in the clozapine and olanzapine groups is presented in Fig. 6, while Fig. 7 shows the distribution of binding gene functional elements.

## 4 Experimental Design, Materials and Methods

### 4.1 Animals and sample collection

Thirty-six female Sprague-Dawley rats (200g- 220g) were purchased from the Animal Resource Centre (Perth, WA, Australia). After one week of environmental adaption, the rats were randomly assigned into three groups: olanzapine (3 mg/kg, b.i.d, orally, Zyprexa, Eli Lilly, Indianapolis, USA), clozapine (20mg/kg, b.i.d, orally, Clozaril, Novartis, Turkey), and vehicle (equivalent sweet cookie dough pellet without drugs) with 12 animals per group. The antipsychotic drugs were mixed into 300mg sweet cookie powder (30% cornflour, 30.9% sucrose, 15.5% casein, 8.4% minerals, 6.4% fibre, 6.3% gelatine, 1.6% vitamins) and appropriate water. Each animal was treated orally twice a day (8:00 am and 8:00 pm, respectively) for nine weeks, as described previously [8]. During the treatment, rats were observed to completely consume drugs pellet. Since the rats avoided taking the cookie pellet with clozapine after 3 days’ treatment, clozapine was delivered using a 1 ml syringe via the mouth, followed by an equivalent sweet cookie dough pellet without drugs to ensure the same amount of cookie pellets was consumed. All animals were housed individually in a temperature-controlled room (22°C) with a 24-hour lighting cycle (lights on 07:00-19:00), and allowed free access to water and standard laboratory chow diet (containing 3.9kcal/g, 10% fat, 16% protein and 74% carbohydrate) throughout the experiment. Body weight, food intake and water consumption were recorded every week. Two hours after the final treatment, all fasting rats were sacrificed by carbon dioxide asphyxiation. Liver tissues were dissected and frozen in liquid nitrogen immediately.

### 4.2 Chromatin immunoprecipitation coupled with next-generation sequencing (ChIP-Seq)

EpiQuik™ Tissue Methyl-Histone H3-K4 ChIP Kit (P-2009-48, Epigentek, East Farmingdale, New York, USA) and EpiQuik™ Tissue ChIP Kit (xy-2003, Epigentek, East Farmingdale, New York, USA) were used to perform ChIP experiments for H3K4me2. According to the manufacturer's instructions, 40 mg of liver tissue was cut into small pieces and cross-linked using 1mL 1% formaldehyde at room temperature for 20 minutes, then was homogenized using Precellys®24 homogenizer (Bertin Technologies, Montigny-le-Bretonneux, France). The chromatin DNA was sheared to an average fragment length of 200–1,000 bp using a Branson 450 Digital Sonifier (Branson, Brookfield, CT, USA) at 20% amplitude with 7 pulses of 18 seconds each. The sheared DNA was diluted with an equal volume of ChIP dilution buffer, then transferred to a strip well pre-incubated with ChIP-grade anti-dimethyl-H3-K4 and anti-normal mouse IgG (negative control) for 60 minutes at room temperature. The cross-linked DNA was then released with 40 µL of release buffer containing 1 µL of Proteinase K and reversed with 40 µL of reverse buffer in a 65°C dry bath for one and a half hours. A spin column was used to purify 20 µL of the DNA. The ChIP DNA was sent to Novogene Co. (Hong Kong) for DNA paired-end library construction and high-throughput sequencing using an Illumina Hiseq2500 50SE sequencer (llumina, San Diego, CA, USA).

### 4.3 Quality control (QC)

To ensure the accuracy and reliability of the sequenced data, quality control (QC) was performed at each step, including sample testing, library preparation, and sequencing. The raw reads from the Illumina HiSeqTM X TEN (llumina, San Diego, CA, USA) also underwent QC to determine their suitability for subsequent analysis. The filtered sequence reads (raw reads) were cleaned through the following three filtering processes: (1) Removing reads containing adapters, (2) Removing reads with N > 10% (N represents bases that cannot be determined), and (3) Removing reads with low-quality bases (Qscore ≤ 5) that constitute over 50% of the total bases.

Adapter sequences:

5′Adapter:
5′AATGATACGGCGACCACCGAGATCTACACTCTTTCCCTACACGACGCTCTTCCGATCT 3′
3′Adapter (The underlined 6bp bases is Index):5′GATCGGAAGAGCACACGTCTGAACTCCAGTCACATCACGATCTCGTATGCCGTCTTCTGCTTG3′

### 4.4 Bioinformation analysis

The Burrows-Wheeler Aligner software was used to map the clean data to the Rattus norvegicus reference genome Rnor_6.0 (http://asia.ensembl.org/Rattus_norvegicus/Info/Index). Only uniquely mapped reads without mismatches were retained for further analysis.

## Limitations

This high throughput data set did not provide the expression levels of target genes or proteins. In addition, it was not supported by ChIP-PCR.

## Ethics Statement

The Animal Ethics Committee at University of Wollongong approved all experimental procedures using animals (AE12/26), according to the Australian Code of Practice for the Care and Use of Animals for Scientific purposes.

## Credit Author Statement

**Yueqing Su**: Conceptualization, Data curation, Formal analysis, Funding acquisition, Investigation, Methodology, Validation, Visualization, Writing –original draft, Writing –review & editing. **Jiamei Lian:** Conceptualization, Formal analysis, Funding acquisition, Investigation, Methodology, Validation, Writing –review & editing. **Chao Deng**: Conceptualization, Funding acquisition, Methodology, Project administration, Resources, Writing –review & editing.

## Data Availability

NCBIDataset of histone H3K4me2 modified genes in the liver of female Sprague-Dawleyrats with chronic antipsychotic drugs of olanzapine or clozapine (Original data).
